# General and violent recidivism of former forensic psychiatric patients in Finland

**DOI:** 10.3389/fpsyt.2023.1157171

**Published:** 2023-06-29

**Authors:** Ilkka Ojansuu, Antti Latvala, Hannu Kautiainen, Jonas Forsman, Jari Tiihonen, Markku Lähteenvuo

**Affiliations:** ^1^Department of Forensic Psychiatry, Niuvanniemi Hospital, University of Eastern Finland, Kuopio, Finland; ^2^Institute of Criminology and Legal Policy, University of Helsinki, Helsinki, Finland; ^3^Primary Health Care Unit, Kuopio University Hospital, Kuopio, Finland; ^4^Folkhälsan Research Center, Helsinki, Finland; ^5^Department of Clinical Neuroscience, Karolinska Institutet, Stockholm, Sweden; ^6^Swedish National Board of Forensic Medicine, Stockholm, Sweden; ^7^Centre for Psychiatry Research, Department of Clinical Neuroscience, Karolinska Institutet, Stockholm, Sweden; ^8^Stockholm Health Care Services, Region Stockholm, Stockholm, Sweden

**Keywords:** recidivism (repeat offense), forensic psychiatry, treatment time, crime, schizophrenia

## Abstract

**Background:**

Forensic psychiatric care in Finland is provided to individuals who have committed a crime due to a serious mental disorder and are in need of psychiatric care. The reconviction (recidivism) rates for this patient group vary in time and between countries, likely due to different treatment practices and requirements for forensic care.

**Materials and methods:**

We set out to study criminal recidivism in a national cohort of all patients released from forensic psychiatric care in Finland between 1999 and 2018. National registries were used to identify the patients and gain information on their criminal sentences. Forensic psychiatric examinations were used to record demographic information for the cohort. The cohort was followed up from hospital discharge to the end of 2019.

**Results:**

We identified a total of 501 patients who were released from forensic psychiatric care (mean age: 46.6 years [SD 13.4), 434 (86.6%) were male). The mean and median times spent in treatment for the cohort was 10.0 years [SD 6.5] and 8.7 years, respectively. 91% of the patients had schizophrenia spectrum disorder (F2*), and 63.5% had a substance use disorder. A total of 83 patients (16.6%) committed any crime after being released from care, and the mean time to recidivism was 3.8 years. The recidivism rate was 2015 per 100,000 person years. A total of 48 patients (9.6%) committed a violent crime. The mean time to violent recidivism was 4.2 years. The violent recidivism rate was 1,083 per 100,000 person years. A longer duration of treatment was associated with a decreased risk of general recidivism (HR 0.95, 95% CI 0.90 to 1.00, *p* = 0.05). Factors associated with higher recidivism were male sex, having a comorbid substance use disorder and younger age at discharge.

**Conclusion:**

The recidivism rate in Finland was markedly lower than has been previously reported for other Western countries, and the mean duration of treatment was also longer. A longer treatment time may reduce the risk of criminal recidivism in forensic psychiatric patients. The results suggest, as previous studies have found, that more effort is indicated on the treatment of substance abuse.

## Introduction

One of the main goals of forensic psychiatric treatment is to reduce the risk of recidivism of crime. The processes of forensic psychiatric care vary from country to country. In Finland, only individuals with a psychotic disorder can be committed to forensic psychiatric care. Unlike in some other countries, individuals with disorder such as bipolar or personality disorders are sentenced to prison instead of forensic psychiatric care regardless of the severity of the condition as long as it does not reach the stated care giving condition–psychosis. As the criteria and processes for forensic psychiatric care are different from country to country, and may vary in time, recidivism rates may not be comparable, and treatment results may not be generalizable between countries. There are several studies published on the risk of recidivism both from Finland as well as other countries, but many of them have small sample sizes, or are already outdated.

Several studies have looked at recidivism risk in Finland during the last decades. In 1950, there was no violent recidivism among 55 patients followed up for more than 10 years (1). In early 1970’s there was only one violent recidivistic crime among 49 patients followed on average for over 7 years (2). In contrast, among 215 patients released from care between 1978 and 1987 there were 14 violent recidivistic crimes, which happened on average 1 year and 7 months after discharge (3). The authors of the study speculated that the main reason behind the increase in violent recidivism could be the abolition of involuntary community care from the Finnish legislation in 1978 (3). In 1990, out of 37 released patients, 2 (5.4%) reoffended during a 1.5 years follow-up (4).

In international studies recidivism rates have varied even more, partly accounted by differences in forensic psychiatric populations, their comorbidities (such as substance use disorders), as well as treatment interventions (5). In a meta-analysis from 2016 of adverse outcomes for forensic psychiatric patients after discharge, crude reoffending rates ranged from 0 to 24,244 per 100,000 person-years (6). The pooled estimate for patients released from forensic care was 4,484 per 100,000 person-years (95% CI 3679–5,287), with very high heterogeneity (*I*2 = 95, 95% CI 94–96%) (6). For violent recidivism, the crude reoffending rates ranged from 273 per 100,000 person-years to 8,403 per 100,000 person-years, with a pooled estimate of 3,902 (95% CI 2671–5,187) with substantial heterogeneity (*I*2 = 97, 95% CI 96–98%) (6).

In a previous Swedish study on forensic patients, young age, earlier convictions, substance use and personality disorder without the presence of psychosis were associated with general recidivism (7). Further, in another more recent Swedish study, shorter treatment duration was significantly associated with greater probability of criminal recidivism after discharge among: men; patients with psychosis; patients without a history of substance use disorder; and among patients whose sentences did not include special court supervision (court-conducted discharge assessment) (8). In other studies including forensic patients in Great Britain and Canada, comorbid personality disorder and substance use disorder were associated with risk of general recidivism (9, 10). However, in a recent meta-analysis of 30 studies, neither age, geographical region, type of index offense, duration of admission or history of in-patient psychiatric treatment were associated with risk of general recidivism (6). In addition, in the same meta-analysis, among 15 studies related to violent recidivism, diagnosis was not associated with risk of violent recidivism.

Here we present a study on recidivism risk for patients released from forensic care in Finland, with over 500 individuals and a follow-up time of up to 20 years. In our study, we set out not only to calculate recidivism risks, but also to decipher the factors associated with increased risk of general and violent recidivism.

## Materials and methods

### Cohort and data collection

The study population consists of all patients committed to forensic psychiatric care in Finland and released during 1999–2018, collected from the archive of the National Institute for Health and Welfare (THL). Currently, there are about 450 beds in governmental forensic psychiatric hospitals, but only about 230 of them are occupied by forensic patients and the rest by general psychiatric difficult to treat patients, in addition to which some forensic patients are treated in community hospitals (11). About 30–35 offenders are admitted to forensic psychiatric care during each year in Finland (11). The treatment time consisted of all compulsory care, which included both in-hospital treatment as well as a possible supervision period of compulsory out-of-hospital treatment. Not all patients had this supervised outpatient care, which in Finland can last for 6 months at a time (but there can be multiple periods) and during which time the forensic patients are still legally considered to be in in-hospital care and can be returned to the hospital if needed. The follow-up of patients started after release from treatment (end of both hospital treatment and the possible supervision period) and lasted until the end of 2019. Release from compulsory care in Finland is determined by the mental health act (“Mielenterveyslaki”) and has to occur when compulsory care is not anymore necessary (e.g., the patient’s psychiatric condition is deemed to be stabilized and the risk for reoffending is assessed as being sufficiently low) (11). After the forensic psychiatric status of a patient has ended, there is no mandatory outpatient treatment, although most patients are advised to attend voluntary outpatient care. The Finnish Forensic Psychiatric system has been detailed in a previous publication (11).

In Finland, only individuals who are charged with a crime and are diagnosed to have a psychotic disorder in a forensic psychiatric examination can be committed to forensic care. Individuals who have committed a crime may request a mental state examination (regardless of crime) and one may be mandated by the court if an individual is charged for a crime for which the sentence may exceed one year in prison. The age criminal liability is 15 years in Finland. Many patients have comorbid personality disorders and substance use disorders, but these disorders in themselves are not sufficient to warrant commission to forensic care. Thus, all patients included in the data had a psychotic disorder, mostly (91%) in the schizophrenia spectrum (ICD-10: F20-29). Of these, 69% had schizophrenia (F20.x), 13% had a delusional disorder (F22.x) and 12% had a schizoaffective disorder (F25.x). The other most common psychiatric disorder groups were psychotic mood disorders (F30–39) in 4% and organic brain syndromes (F0–9) in 4%. The forensic psychiatric examination notes were used to record substance use disorder comorbidities (SUD). The examinations were also reviewed by one of the authors (Dr. Ilkka Ojansuu) in order to identify any SUDs that were described in the statement but for which no diagnosis was given. Any patient with evidence of substance dependence syndrome or harmful use (ICD-10: F1x.1–F1x.2) was counted as having an SUD regardless of the substance. Based on the collected data, the patients were divided into two groups based on whether they were suffering or not suffering from SUD at the time of the forensic psychiatric examination.

Information on recidivism was gathered from the database of the Institute of Criminology and Legal Policy at the University of Helsinki, which holds information on all criminal convictions in Finland between 1999 and 2019, provided by the Legal Register Centre. Thus, only convictions were considered recidivism.

Mortality data were collected from the register of Statistics Finland in order to censor follow-up on death. The Finnish personal identity number was used to link individuals between registers.

### Statistical analyses

Data are presented as means with standard deviation (SD) or as counts (n) with percentages (%). The Kaplan–Meier method was applied to estimate the cumulative incidence of crimes and the permutation type log-rank test was used to analyze the statistical differences between survival curves. Follow-up was censored at death or first recidivistic violent (when calculating violent recidivism) or non-violent crime (when calculating all recidivism). Cox proportional hazards regression was used to estimate the crude and adjusted hazard ratios (HR) and their 95% confidence intervals (CIs). Age at discharge from hospital and gender were used as covariates in these models when appropriate. *p*-values of 0.05 or lower were considered statistically significant. p-values were not corrected for multiple comparisons. All analyses were performed using STATA software, version 17.0 (StataCorp LP, CollegeStation, TX).

### Ethical considerations

This study was purely registry based and no contacts were made with the subjects of the study. The study was approved by THL and by Statistics Finland. The ethical review for the project was conducted by the Finnish Institute for Health and Welfare prior to granting access to the registry data. All data were analyzed in pseudonymized form.

## Results

**Figure 1:**
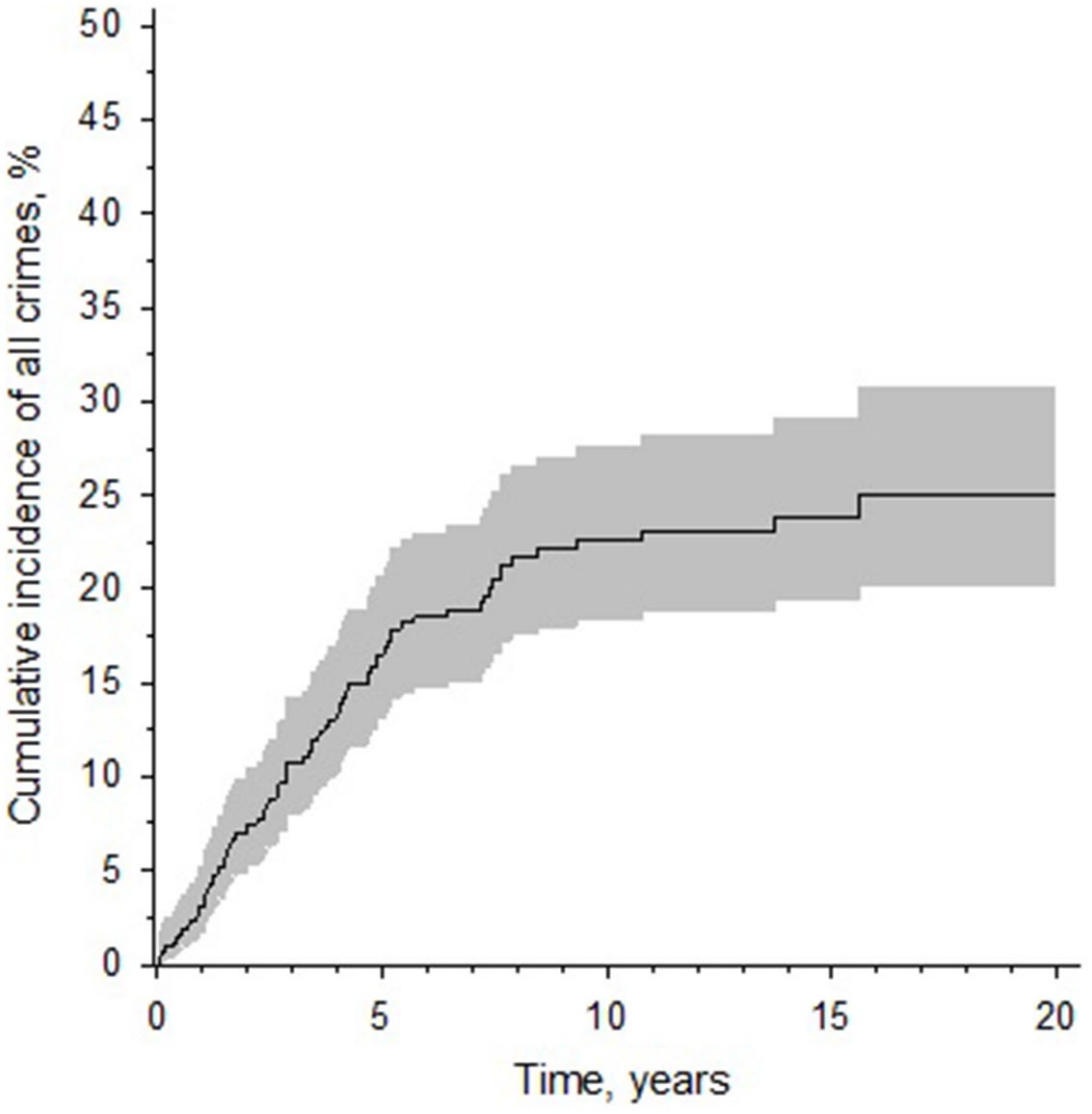
A Kaplan–Meier curve displaying the cumulative incidence of recidivism after release from forensic psychiatric care in Finland (for individuals released from care during 1999–2019). *Y*-axis denotes the cumulative incidence of general recidivism in percentages. *X*-axis denotes follow-up time. The gray area represents the 95% confidence intervals.

During the follow-up period 501 patients were released from forensic psychiatric care. Of the released patients, 434 were male and 67 were female. The mean treatment time for the whole cohort was 10.0 years (range 0.3–34.1 years), 10.2 (range 0.3–34.1) years for males and 8.8 (range 0.3–24.6) years for females. The median treatment time was 8.7 years, 8.8 for males and 7.4 for females. The patients were followed from release from hospital to either recidivism, death or the end of year 2019, whichever came first. The total follow-up time for the cohort was 4,119 person years (pyrs). The mean follow-up time was 8.2 years (SD 5.8 years). During the follow-up, 83 patients (16.6%, 81 male, 2 female) had committed any recidivistic crime and of them 48 (9.6%, 46 male, 2 female) a violent recidivistic crime. For 30 of the reoffenders, their first reoffense was a violent crime and for 53 a non-violent crime. The mean time from release to recidivism was 3.8 (0.1–15.6) years and median time 2.9 years. The risk of general recidivism was the highest during the first 5 years after release and significantly decreased after 10 years from release (Figure 1). A longer duration of treatment was associated with a decreased risk of general recidivism (HR 0.95, 95% CI 0.90 to 1.00, *p* = 0.05). The cumulative incidence of general recidivism for 5, 10 and 15 years for the whole cohort and separated by sex are shown in Table 1.

**Table 1:** The cumulative incidence of recidivism after 5, 10, and 15 years of release from forensic psychiatric care, separated by sex.

Time from discharge	Recidivism % for any crime		
	Males, % (95% CI)	Females, % (95% CI)	Whole cohort, % (95% CI)
5 years	16.5 (13.1 to 20.7)	0.0	14.2 (11.2 to 17.9)
10 years	22.6 (18.4 to 27.6)	3.1 (0.4 to 20.2)	19.8 (16.1 to 24.3)
15 years	23.9 (19.5 to 29.2)	3.1 (0.4 to 20.2)	21.0 (17.1 to 25.7)

**Figure 2:**
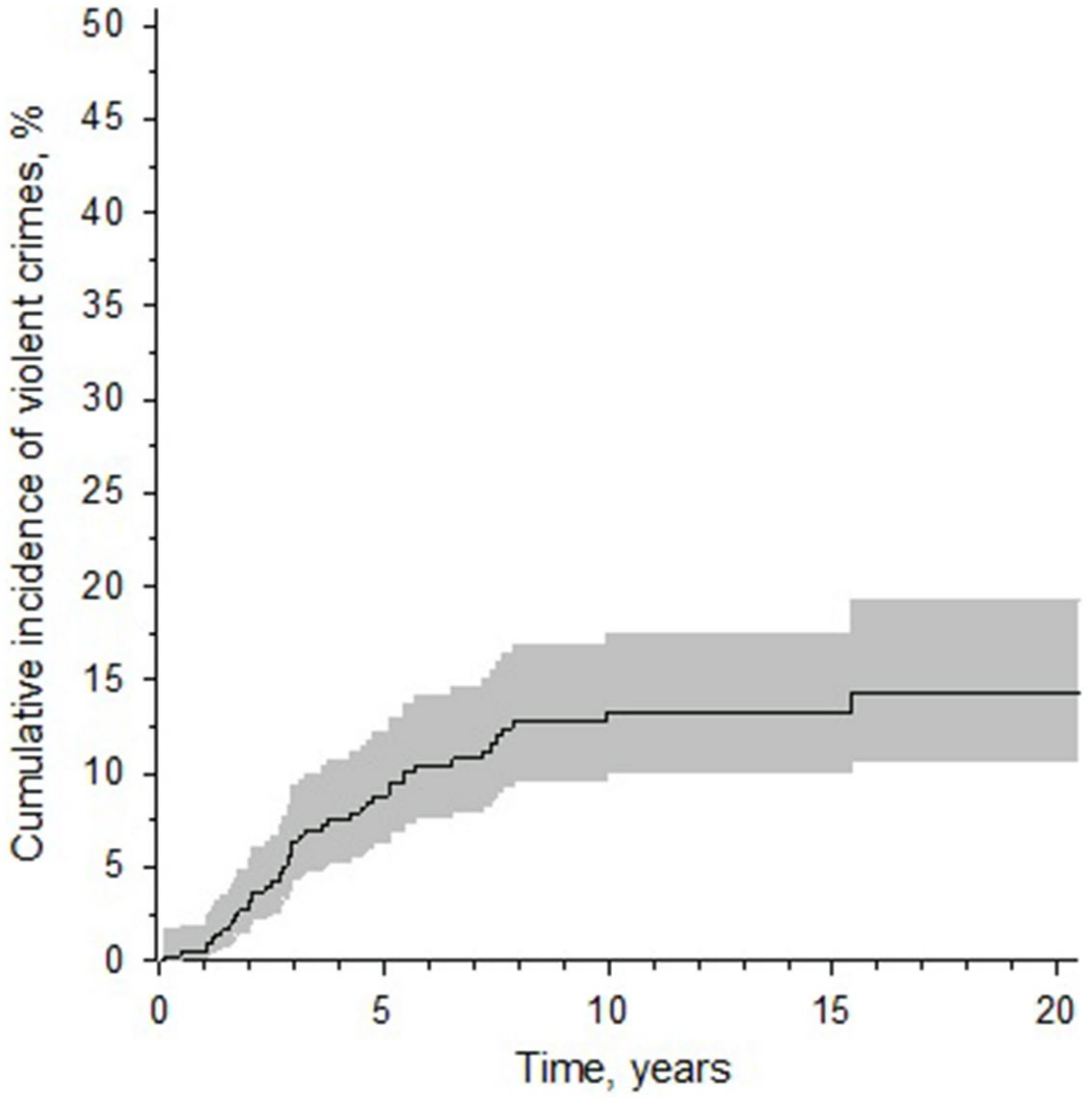
A Kaplan–Meier curve displaying the cumulative incidence of violent recidivism after release from forensic psychiatric care in Finland (for individuals released from care during 1999–2019). *Y*-axis denotes the cumulative incidence of violent recidivism in percentages. *X*-axis denotes follow-up time. The gray area represents the 95% confidence intervals.

Among sentenced reoffenders the mean time from release to violent recidivism was 4.2 (0.1–15.6) years and median time 2.9 years. The risk of violent recidivism was also highest during the first 5 years after release and had significantly decreased after 10 years from release (Figure 2). After 15 years no violent recidivism was noted. The cumulative incidence of violent recidivism for 5, 10, and 15 years for the whole cohort and separated by sex are shown in Table 2.

**Table 2:** The cumulative incidence of violent recidivism after 5, 10 and 15 years of release from forensic psychiatric care, separated by sex.

Time from discharge	Recidivism % for violent crime		
	Males, % (95% CI)	Females, % (95% CI)	Whole cohort, % (95% CI)
5 years	8.8 (6.3 to 12.2)	0.0	7.6 (5.4 to 10.5)
10 years	13.3 (10.0 to 17.5)	3.1 (0.4 to 20.2)	11.8 (8.9 to 15.6)
15 years	13.3 (10.0 to 17.5)	3.1 (0.4 to 20.2)	11.8 (8.9 to 15.6)

**Figure 3:**
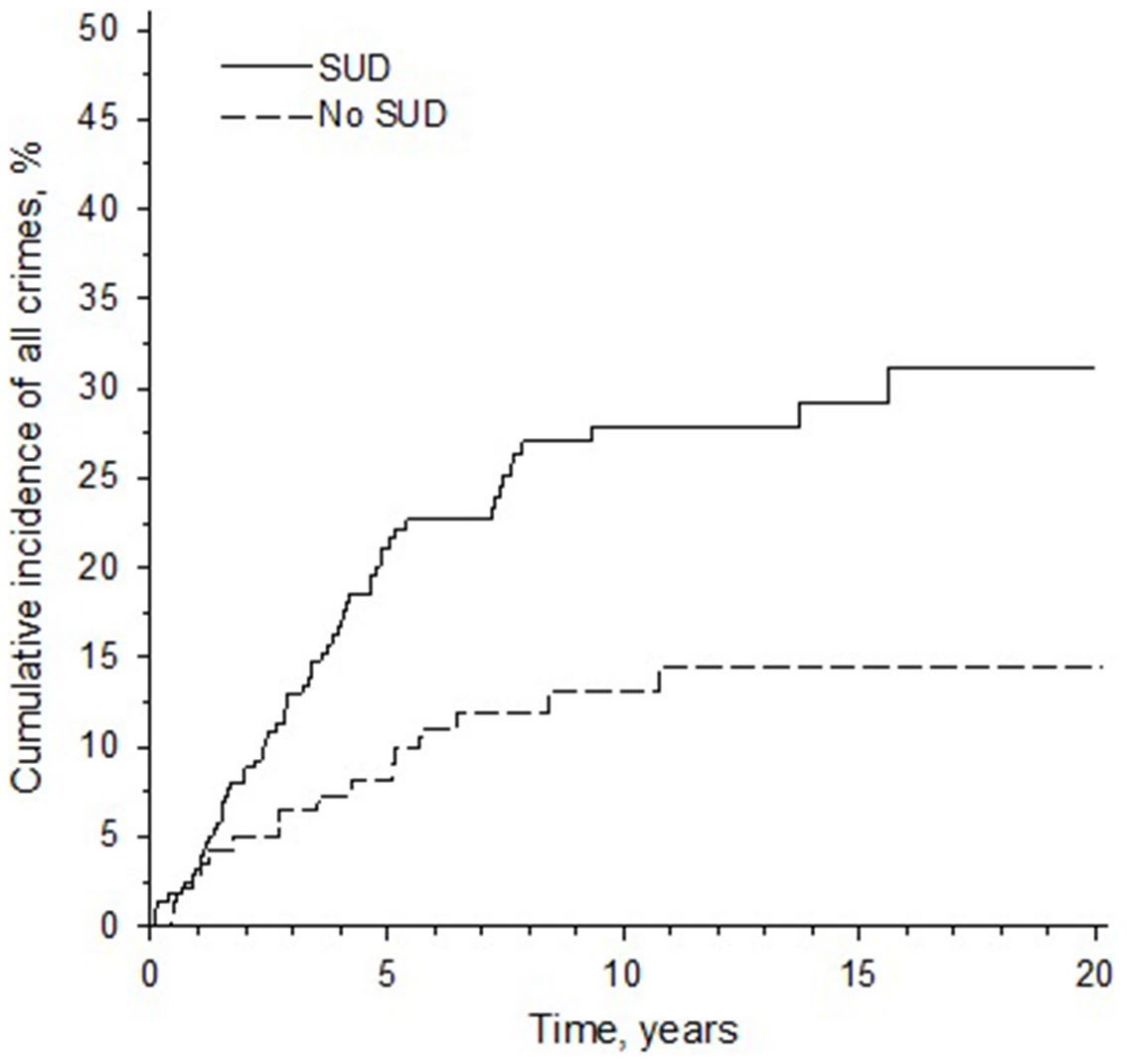
A Kaplan–Meier curve displaying the cumulative incidence of recidivism for patients with and without a substance use disorder (SUD). *Y*-axis denotes the cumulative incidence of general recidivism in percentages. *X*-axis denotes follow-up time.

The mean age of the patients was 47 years at time of release. The mean age at release for those who did not commit a recidivistic crime was 49 years and for those who did 38 years. Older age at release was significantly associated with a decreased risk of general recidivism (HR 0.94, 95% CI 0.91 to 0.96, *p* < 0.001). Of the cohort, 63.5% (65.9% of males, 47.8% of females) had a comorbid substance use disorder according to ICD-10 criteria (either dependence or harmful use) at time of forensic psychiatric examination. The patients with a SUD had a significantly higher rate of general recidivism rate (HR 2.46, 95% CI 1.46 to 4.15, *p* < 0.001), especially during the first 5 years after release from hospital (Figure 3) than patients without a SUD. The risk of recidivism was especially high for those with a SUD whose treatment time had been less than 8 years (Table 3).

**Table 3:** Risk of recidivism per treatment time and having vs. not having a substance use disorder diagnosis at time of forensic psychiatric examination.

Treatment time, years	No SUD			SUD		
	N	Events	HR (95% CI)	N	Events	HR (95% CI)
*(A) Whole cohort*						
0–5.0	57	10	1.00 (Reference)	60	17	2.48 (1.13–5.47)
5.5–8.0	47	4	0.59 (0.18–1.87)	89	27	2.34 (1.13–4.88)
8.5–12.5	31	4	1.18 (0.36–3.81)	89	12	1.28 (0.54–3.02)
13.0+	48	1	0.38 (0.05–3.06)	80	8	1.54 (0.58–4.07)
*(B) Men*						
0–5.0	44	8	1.00 (Reference)	55	17	2.91 (1.24–6.82)
5.5–8.0	35	4	0.83 (0.25–2.75)	79	27	2.96 (1.33–6.58)
8.5–12.5	30	4	1.26 (0.37–4.24)	77	12	1.59 (0.64–3.98)
13.0+	39	1	0.50 (0.06–4.17)	75	8	1.69 (0.61–4.72)

(A) Whole cohort (B) Males. HR = Hazard ratio. Results are corrected for age at discharge.

The recidivism rate per 100,000 person years for the whole cohort for general recidivism (any crimes) was 2015 (95% CI 1605 to 2,497) and 2,302 (95% CI 1828 to 2,861) for males and 333 (95% CI 40 to 1,203) for females. For violent crime 1,083 (95% CI 799 to 1,436) for the whole cohort and 1,201 (95% CI 40 to 1,203) for males and 333 (95 CI 40 to 1,203) for females. Male sex was associated with a markedly increased risk of general recidivism (HR 7.18, 95% CI 1.75 to 29.23, *p* < 0.001). When considering the effect of substance use disorder, the recidivism rate per 100,000 person years for general recidivism was 1,091 (95% CI 657 to 1704) for individuals without SUD and 2,692 (95% CI 2073 to 3,437) for those with SUD (IRR 2.65, 95% CI 1.58 to 4.44, p < 0.001). For violent recidivism, the rate was 604 (95% CI 302 to 1,081) for individuals without SUD and 1,417 (95% CI 997 to 1952) for individuals with SUD (IRR 2.42, 95% CI 1.23 to 4.77, *p* < 0.011).

## Discussion

This study found that of the 501 Finnish forensic psychiatric patients released from care between 1999 and 2018, 17% generally reoffended and 10% violently reoffended during a mean follow-up of 8 years. The general recidivism rate is low when compared to released prisoners in Finland, as 59.3% of released prisoners reoffended within 5 years of release with a crime for which they were sentenced either time in prison or community service (12), but somewhat comparable, although on the lower side, to other international forensic samples reporting recidivism rates between 16 and 49% (13–18). However, percentual reoffending is highly skewed by follow-up time, so a more comparable measure is recidivism rate per 100,000 person-years. A recent meta-analysis reported that for forensic psychiatric patients the pooled crude reoffending rate was 4,484 per 100,000 person-years for any recidivism and 3,902 per 100,000 person-years for violent recidivism (6). In our study the comparable numbers were 2015 for any recidivism and 1,083 for violent recidivism. However, drawing conclusions from these comparisons is difficult, since there are marked differences between forensic psychiatric populations, for example with regard to diagnoses that may lead to forensic care, as well as treatment protocols between countries.

In regard to the general population of patients with schizophrenia, in a national registry study in Finland, 59% of inpatients with schizophrenia released from a general psychiatric hospital had a relapse requiring hospitalization during a median 14 year follow-up (maximum follow-up time 20 years) (19). As the recidivism rate noted in our current study is lower, it would imply that either the relapse rate in forensic patients is lower or, more plausibly, that not every relapse requiring hospitalization leads to recidivism even among forensic patients. However, we did not have information on the relapses of our cohort, so more data would be needed to decipher the risk between relapse and recidivism.

Even though the recidivism rate in Finland was well below the international average reported on the cited meta-analysis (6), it was still markedly higher than the rates published in Finland from the previous decades (1–4). The most notable change seems to have happened in the end of 1970s when compulsory outpatient forensic care was abolished, and patients were no longer supplied with lay guardians to look after them. Since the overall incidence of the most severe violent crimes in general has decreased in Finland during the last 30 years, the changes in compulsory outpatient care may to some extent explain the rise in recidivism rates after that period, as they are not explained by a general rise in violent crime (20). They are also not explained by reductions in treatment times in forensic care, as the mean treatment duration for forensic patients was 3.7 years in the 1950’s (1) and 4.2 years in the largest forensic hospital in Finland in 1988 (3), but 10 years in our study. One explaining factor is likely to be the longer follow-up time in the current study, as especially percentual recidivism is highly affected by follow-up time, since incidents accumulate over time.

We discovered in our study some factors that were related to risk of recidivism. The most notable factor that was associated with increased risk of both general and violent recidivism was having a substance use disorder, a risk factor also observed in previous studies (7–10). The effect of SUDs is especially clear, when comparing the rates of reoffending per 100.000 person years, as the rates were 2,692 for all recidivism and 1,417 for violent recidivism for those with SUD and 1,417 for all recidivism and 604 for violent recidivism for those without SUD. Thus, the rates were almost two-fold for any recidivism and over two-fold for violent recidivism for those with SUD. SUD is a risk factor that is often taken into account in risk assessment and management protocols, but the results clearly show that at least in Finland we are unable to mitigate the risks of recidivism related to SUDs in our treatment protocols.

Factors associated with decreased risk of general recidivism were a longer treatment duration and higher age at discharge, which are naturally interconnected. As the treatment times for forensic patients in Finland are long (mean time 10.0 years in our sample) as compared to international samples (3 years in the meta-analysis by Fazel et al.), the long treatment duration is likely to account for at least part of the lower rate of recidivism, although the interconnected age did not appear to be statistically associated with risk of recidivism in the previous meta-analysis (6). The effect of treatment time on general recidivism was especially strong for those with a SUD. Thus, patients with a SUD may benefit from extended treatment times, as forensic hospitals focus not only on treating psychotic disorders, but also substance use disorders. Patients with substance use disorder often also have antisocial traits, which have been shown to reduce with age, which may also in part explain for the reduction in recidivism with an older age of release (21).

Although sex in itself is a non-modifiable risk factor, some of the effects mediated by sex may well be. In our sample, substance use disorders were more prevalent in males than in females, but the difference in prevalence was not enough to alone explain for the increased recidivism observed in males. More research is needed in the future to focus on the traits associated to male sex that may mediate the increased risk of recidivism observed, in order to develop strategies to reduce risk of recidivism. On the other hand, female recidivism seems to be very rare and may indicate that forensic treatment times for females in Finland are too excessive in terms of preventing recidivism.

In conclusion, our results suggest that longer forensic psychiatric treatment is associated with reduced general recidivism, a phenomenon especially well seen in patients with a SUD. Higher age at discharge was also associated with lower risk of general recidivism, which may be related longer treatment times (or vice versa). Nevertheless, patients with a SUD may benefit from longer forensic treatments and forensic treatment needs to focus more also on the treatment of SUDs from a risk reduction perspective. Female recidivism was scarce and treatment times for females in Finland may be excessive.

### Strengths and weaknesses

A number of issues need to be considered when interpreting the results of our study. Our study had a large and inclusive cohort with a considerable follow-up time. However, only patients with psychotic disorders are committed to forensic care in Finland, and thus results are not generalizable to countries where other disorders also enable forensic care. Unfortunately, we were not able to obtain information on what kind of care was provided to the patients after their discharge. Thus, we could not take into account the effects of treatment contacts, living arrangements, use of medication and other treatments when assessing risk of recidivism. These are factors which likely have a significant effect on recidivism and further research on these factors is warranted.

## Data availability statement

The original contributions presented in the study are included in the article/supplementary material, further inquiries can be directed to the corresponding author.

## Ethics statement

Ethical review and approval was not required for the study on human participants in accordance with the local legislation and institutional requirements. Written informed consent for participation was not required for this study in accordance with the national legislation and the institutional requirements.

## Author contributions

IO: conception and design of the work, collecting, analysis and interpretation of data, and drafting of the manuscript. AL: analysis and interpretation of data and critical revising of the manuscript. HK: statistical analysis, interpretation of data, and critical revising of the manuscript. JF: design and interpretation of data and critical revising of the manuscript. JT: acquisition, analysis and interpretation of data and critical revising of the manuscript. ML: design of the work, analysis and interpretation of data, and drafting of the manuscript. All authors contributed to the article and approved the submitted version.

## Funding

The study was funded by the Finnish Ministry of Social Affairs and Health, through the developmental fund for Niuvanniemi hospital.

## Conflict of interest

The authors declare that the research was conducted in the absence of any commercial or financial relationships that could be construed as a potential conflict of interest.

## Publisher’s note

All claims expressed in this article are solely those of the authors and do not necessarily represent those of their affiliated organizations, or those of the publisher, the editors and the reviewers. Any product that may be evaluated in this article, or claim that may be made by its manufacturer, is not guaranteed or endorsed by the publisher.
